# Retention rates of infliximab and tocilizumab during a 3-year period in a Brazilian hospital

**DOI:** 10.1590/S1679-45082013000400015

**Published:** 2013

**Authors:** Ricardo Prado Golmia, Morton Aaron Scheinberg

**Affiliations:** 1 Hospital Israelita Albert Einstein, São Paulo, SP, Brazil.

**Keywords:** Biological agents, Arthritis, rheumatoid/drug therapy, Antibodies, monoclonal/therapeutic use

## Abstract

**Objective:**

To compare the efficacy and the period of use of tocilizumab and infliximab during treatment of rheumatoid arthritis patients.

**Methods:**

The period of use of two biologics with different mechanisms of action were compared in treatment of rheumatoid arthritis patients.

**Results:**

Both medications showed efficacy, but the period of use with no loss of efficacy was longer in patients receiving tocilizumab when compared to infliximab.

**Conclusion:**

Tocilizumab maintains a period of use significantly longer as compared with infliximab in patients with rheumatoid arthritis treated at a single organization.

## RESUMO

**Objetivo:**

Comparar a eficácia e período de uso de tocilizumabe e infliximabe no tratamento de pacientes com artrite reumatoide.

**Métodos:**

Foi comparado o tempo de uso de dois biológicos com diferentes mecanismos de ação no tratamento de pacientes com artrite reumatoide.

**Resultados:**

Ambos os biológicos se mostraram eficazes, mas o tempo de uso sem perda de eficácia foi maior com tocilizumabe quando comparado ao infliximabe.

**Conclusão:**

Tocilizumabe mantém um período de uso significativamente maior do que infliximabe em pacientes com artrite reumatoide tratados em uma única instituição.

## INTRODUCTION

Biological disease-modifying anti-rheumatic drugs (biological agents) are the standard treatment for rheumatoid arthritis (RA). Significant amount of data is now available showing improvement in signs and symptoms of RA patients with both early and established disease^(1)^. Some patients with RA on use of biological agents are compelled to stop administration of these drugs or switch due to lack of efficacy and development of adverse events^(2)^. During the real life practice, it became apparent to one of us (MS) that discontinuation or switch of biological therapy was more frequent in patients receiving infliximab than tocilizumab. We therefore looked retrospectively during a 3-year period the retention rate of patients receiving either infliximab or tocilizumab at *Hospital Israelita Albert Einstein* and the results observed are described in this article. Since this was not part of an ongoing protocol, the specific metrics of disease activity were not routinely recorded for almost half of the patients included in this report.

## OBJECTIVE

To compare the efficacy and the period of use of tocilizumab and infliximab during treatment of rheumatoid arthritis patients.

## METHODS

Patients with diagnosis of RA were seen by two rheumatologists at their respective clinics at *Hospital Israelita Albert Einstein*, in the city of São Paulo. Patients with inadequate response to disease-modifying drugs were assigned to receive either infliximab or tocilizumab, during the period from September 2009 to September 2012, and were included in this report. Treatment failure was determined when patients showed no improvement in signs and symptoms or no reduction in DAS28 greater than 1.0. The adverse events were defined as clinical manifestations that led to treatment discontinuation in spite of demonstrating efficacy. Secondary treatment was defined as patients having received previous biologic therapy.

This paper was approved by the Ethical Committee of *Associação de Assistência à Criança Deficiente de São Paulo*, protocol # 72/2011.

### Statistical analysis

Comparisons of qualitative variables were performed using the Khi2 test

## RESULTS

Table 1 presents the results obtained with the use of tocilizumab. Out of a total of 61 patients, approximately one third (31%) had the medication discontinued due to lack of efficacy, and 8% due to the presence of adverse events. The discontinuation rate was 40% during the period of the study. Treatment failure was more frequent in secondary treatment than in the primary response. Table 2 displays the results with infliximab during the 3-year period. Sixty-eight patients received infliximab and 36% had their medication discontinued due to lack of efficacy after variable periods of administration. Nine patients had infliximab discontinued due to adverse events. In this group, dose titration, defined by shorten period of administration or increasing dosage, was also included if no response was observed. Out of 61 patients receiving tocilizumab, 51 had previously received anti-tumor necrosis factor (TNF). The difference between survival rates between tocilizumab and infliximab was statistically significant (p<0.01). Although 129 patients were included in this report it is possible that patients seen by other rheumatologists at the institution may have received the two biologics but are not reported in this evaluation.

**Table 1:** Retention rates during therapy (2009–2012)

Number of patients	Loss of efficacy	Adverse events
61 (tocilizumab)	18	8
68 (infliximab)	36*	9

* The retention rate of infliximab was significantly higher than the one of tocilizumab (p<0.01).

**Table 2:** Adverse events

Adverse events (tocilizumab)	Number of patients
Urticaria after infusion	2
Hypertension headache	2
Hypotension after infusion (severe)	2
Skin disease (developed lichenoid reaction)	1
Skin disease (herpes zoster)	1
Adverse events (infliximab)	**Number of patients**
Chest pain	1
Infusion reactions	3
Skin infection (herpes zoster)	2
Repeated urine infection	1
Deep fungal infection	1
Tuberculosis	1

## DISCUSSION

Biologicals are an important addition to care of patients with RA. However, there is an increasing knowledge that compliance to the first biological varies much and no standardized methods are available to track persistence and compliance to the initially instituted biological treatment^(3)^. To our knowledge, this is the first report comparing drug retention rates in a single group combining lack of primary and secondary efficacy; in addition to verifying adverse events in two biologicals with different mechanisms of action.

The retention rates significantly differed among patients on use of infliximab when compared to tocilizumab (p<0.01). Occurrence of adverse events that led to discontinuation of treatment did not differ significantly between the two agents although these events were somehow diverse. Patients on infliximab had higher discontinuation rate due to lack of efficacy. There are reports similar to this present study but, unlike ours, they all are based on registries. Nevertheless, the results from these investigations do not differ from those found in our study, showing a rather high rate of discontinuation in patients using infliximab after a certain period of time^(4,5)^. The health insurance companies at the hospital do not authorize payment for dose titration, but it is known that when there is loss of efficacy, increasing the dosage may lead to reversal of resistance^(6,7)^. Retention rates among TNF-blocker users were not compared, although it was reported that infliximab is also associated with decreased retention rates due to infusion or allergic reactions^(8)^. One of the limitations of the present study is the fact that all patients had access to their biological agent through their private health insurance plans, which did not authorize the use of subcutaneous anti-TNF blockers at hospitals. Hence, they precluded access to information on other anti-TNF blockers at the same organization.

Our results observed with tocilizumab retention rates after prolonged use were similar to those reported from Japan, in real life clinical practice, when evaluated retrospectively or compared to infliximab^(6)^.

Recent report from the Swiss^(5)^ registry has also presented results similar to those of the present study. After inadequate response to TNF-alpha blocker, and particularly after primary failure, patients on a non-TNF alpha biological agent had significantly higher drug retention rates. The retention rates for infliximab were significantly lower when compared to although tocilizumab; both agents have different mechanisms of action and showed efficacy in treating RA.

## CONCLUSIONS

In conclusion, we presented a direct comparison of the use of tocilizumab and infliximab at a single institution. The retention rates for infliximab were significantly lower as compared to tocilizumab and both agents were efficacious in treating rheumatoid arthritis, despite having different mechanisms of action.
